# Association of Interleukin-6–174G/C Polymorphism With Ischemic Stroke: An Updated Meta-Analysis

**DOI:** 10.3389/fneur.2021.799022

**Published:** 2022-01-06

**Authors:** Jie Chai, Xian-Ling Cao, Feng Lu

**Affiliations:** ^1^College of Traditional Chinese Medicine, Shandong University of Traditional Chinese Medicine, Jinan, China; ^2^Cardiovascular Internal Medicine, The Affiliated Hospital of Shandong University of Traditional Chinese Medicine, Jinan, China

**Keywords:** IL-6, −174G/C, rs1800795, genetic polymorphism, ischemic stroke

## Abstract

**Background:** Although numerous epidemiological studies have investigated the association between −174G/C(rs1800795) polymorphism in the interleukin-6 (IL-6) gene-stimulatory region and the risk of ischemic stroke (IS), they failed to reach a unified conclusion. The true relationship between −174G/C(rs1800795) polymorphism and IS remains controversial and unclear. Therefore, in this meta-analysis, we aimed to analyze more precisely the association between −174G/C(rs1800795) single-nucleotide polymorphism (SNP) of IL-6 gene and IS in a larger pooled population.

**Methods:** A comprehensive literature search was performed in *PubMed, Web of Science*, and *the Cochrane Central Register of Controlled Trials* until June 30, 2021. A fixed or random-effects model was utilized based on heterogeneity between studies. The odds ratios (ORs) and 95% confidence intervals (Cis) were calculated in the models of allele comparison (G vs. C), homozygote comparison (GG vs. CC) and (GC vs. CC), dominant (GG vs. GC + CC), hyper dominant (GG + CC vs. GC), and recessive (GG + GC vs. CC) to determine the strength of associations.

**Results:** This meta-analysis included 13 case-control studies in 35 articles with 5,548 individuals. Overall, no significant associations between IL-6 −174G/C(rs1800795) and IS were identified (G vs. C:OR [95% CI] = 0.99 [0.81, 1.21], *P* = 0.91; GG + CC vs. GC:0.97 [0.85, 1.11], *P* = 0.66; GG vs. GC + CC: 1.01 [0.81, 1.25], *P* = 0.94; GC vs. CC: OR [95% CI] = 1.01 [0.68, 1.5], *P* = 0.96; GG vs. CC:0.93 [0.57, 1.51], *P* = 0.76; GG + GC vs. CC:0.97 [0.64, 1.47], *P* = 0.89). In the subgroup analyses by ethnicity or HWE *P*-value, there was a statistically significant association between IL-6 −174G/C(rs1800795) polymorphisms and IS in the alleles model; (G vs. C: LogOR [95% CI] = 0.14 [−0.16,.45], *P* = 0.00), homozygote model (GG vs. CC: LogOR [95% CI] = 0.18 [−0.58,.95], *P* = 0.00) and (GC vs. CC: LogOR [95% CI] = 0.2 [−0.46,.85], *P* = 0.00), dominant model (GG vs. GC + CC: OR [95% CI] = 0.02 [−0.72, 0.77], *P* = 0.00), and recessive model (GG + GC vs. CC: OR [95% CI]= −0.17 [−0.86,.52], *P* = 0.00) of the European population and in the dominant model (GG vs. GC + CC: OR [95% CI] = −0.13 [−0.51, 0.24]) of the Asian population. No statistical significance was identified in both six models of HWE *p* ≥ 0.2 group (both *P* ≥ 0.05).

**Conclusion:** This meta-analysis revealed no correlation between IL-6 −174G/C(rs1800795) polymorphism and IS, whereas the subgroup analysis indicated that the relationship between IL-6 −174G/C(rs1800795) polymorphism and IS susceptibility varied significantly according to ethnicity and geography.

## Introduction

Stroke is characterized by high rates of morbidity, mortality, recurrence, and disability. Examples of strokes include ischemic strokes (IS) and hemorrhagic strokes (HS). IS accounts for 70-90% of the incidence of strokes (1). It can cause irreversible neuronal damage in the ischemic area within a few minutes (2). Furthermore, studies indicated that IS is also the second leading cause of preventable deaths and the third leading cause of long-term disability among adults worldwide (3).

The pathogenesis of ischemic stroke (IS) is complex and unclear. Inflammatory factors have been demonstrated to play a fundamental role in the physiology, etiology, and pathology of IS and other brain injury forms (4). Moreover, studies have indicated that inflammation is critical in atherosclerosis occurrence and development, which is common across several IS subtypes when classified according to schemes such as Trial of ORG 10172 in Acute Stroke Treatment. It has also been demonstrated to be linked to an increased susceptibility to stroke and coronary heart disease (4, 5). Not only does inflammation play an important role in IS development caused by atherosclerosis, but Kelly et al. also reported that inflammation could influence different pathogenic subtypes of IS by contributing to a prothrombotic status regardless of the stroke subtype (5, 6). Moreover, embolic strokes of undetermined sources (ESUS) account for one-third of IS. Acampa et al. found that the relationship between AF and ESUS may be mediated by inflammation rather than a simple cause-and-effect mechanism (6). Likewise, they stated that the increased risk of AF in a cryptogenic stroke might be due to inflammation-mediated atrial remodeling and electrical remodeling (7). These studies reveal that inflammation plays an important pathological role in different IS types, especially in cryptogenic strokes inflammation that can also promote atrial cardiopathy, which is a potential new pathogen.

The brain's response to ischemic injury can be regarded as an acute and long-term inflammatory process, characterized by rapid polarization of microglia, production of pro-inflammatory cytokines, and presentation of various leukocyte types into ischemic brain tissue, leading to IS occurrence and development (8). This was confirmed by increased concentrations in pro-inflammatory cytokines in the blood, cerebrospinal fluid in patients, and by studies of animal-induced experiments (9). Therefore, many studies focused on the role of pro-inflammatory cytokines such as tumor necrosis factor-α (TNF-α), interleukin-1 (IL-1), interleukin-10 (IL-10), and interleukin-6 (IL-6) in IS pathogenesis. These studies found that genes, which are cytokines with anti-inflammatory properties, may contribute to IS occurrence and development (9–11).

Therefore, functional polymorphisms of inflammatory genes may influence IS incidence and outcome. The contribution of IL-6 genetic polymorphism to the change in IS IL-6 levels was also reported (12). A part of SNPs identified in the IL-6 gene has a substantial impact on gene expression that can alter plasma levels of IL-6. For instance, the promoter variant (G-572C) can influence the transcription efficiency of IL-6, which may play a role in inflammation-related diseases such as IS. Nevertheless, researchers found that −174G/C(rs1800795) SNP in the promoter region of IL-6 gene did not contribute to the increase of IL-6 level in plasma (12, 13). Many studies investigated the potential association between IL-6 −174G/C(rs1800795) polymorphism and IS. However, these findings are inconsistent, and the sample size of individual studies is statistically insufficient. Therefore, the relationship between SNP −174G/C(rs1800795) in the promoter region of the IL-6 gene and IS remains controversial and unclear.

In this study, a meta-analysis was performed to better evaluate the relationship between IL-6 −174G/C(rs1800795) polymorphism and IS in a larger clustered population.

## Methods

### Search Strategy

We searched *PubMed, Web of Science*, and *the Cochrane Central Register of Controlled Trials* databases for papers associating IL-6 −174G/C polymorphism and IS available by June 30, 2021, without language restrictions using the following search terms:

(“Ischemic Stroke” OR “Ischemic Strokes” OR “Stroke, Ischemic” OR “Ischaemic Stroke” OR “Ischaemic Strokes” OR “Stroke, Ischaemic” OR “Cryptogenic Ischemic Stroke” OR “Cryptogenic Ischemic Strokes” OR “Ischemic Stroke, Cryptogenic” OR “Stroke, Cryptogenic Ischemic” OR “Cryptogenic Stroke” OR “Cryptogenic Strokes” OR “Stroke, Cryptogenic” OR “Cryptogenic Embolism Stroke” OR “Cryptogenic Embolism Strokes” OR “Embolism Stroke, Cryptogenic” OR “Stroke, Cryptogenic Embolism” OR “Wake-up Stroke” OR “Stroke, Wake-up” OR “Wake up Stroke” OR “Wake-up Strokes” OR “Acute Ischemic Stroke” OR “Acute Ischemic Strokes” OR “Ischemic Stroke, Acute” OR “Stroke, Acute Ischemic”) AND (“Interleukin-6” OR “Interleukin 6” OR “IL6” OR “B-Cell Stimulatory Factor 2” OR “B-Cell Stimulatory Factor-2” OR “Differentiation Factor-2, B-Cell” OR “Differentiation Factor 2, B Cell” OR “B-Cell Differentiation Factor-2” OR “B Cell Differentiation Factor 2” OR “BSF-2” OR “Hybridoma Growth Factor” OR “Growth Factor, Hybridoma” OR “IFN-beta 2” OR “Plasmacytoma Growth Factor” OR “Growth Factor, Plasmacytoma” OR “Hepatocyte-Stimulating Factor” OR “Hepatocyte Stimulating Factor” OR “MGI-2” OR “Myeloid Differentiation-Inducing Protein” OR “Differentiation-Inducing Protein, Myeloid” OR “Myeloid Differentiation Inducing Protein” OR “B-Cell Differentiation Factor” OR “B Cell Differentiation Factor” OR “Differentiation Factor, B-Cell” OR “Differentiation Factor, B Cell” OR “IL-6” OR “Interferon beta-2” OR “Interferon beta 2” OR “beta-2, Interferon” OR “B Cell Stimulatory Factor-2” OR “B Cell Stimulatory Factor 2”) AND (“Polymorphism, Genetic” OR “Genetic Variation” OR “Genetic Variations” OR “Variations, Genetic” OR “Variation, Genetic” OR “Diversity, Genetic” OR “Diversities, Genetic” OR “Genetic Diversities” OR “Genetic Diversity” OR “Polymorphisms, Genetic” OR “Genetic Polymorphisms” OR “Genetic Polymorphism” OR “Polymorphism (Genetics)” OR “Polymorphisms (Genetics)”) AND (“174” OR “rs1800795”).

### Study Selection

Citations selected from this initial search were screened and assessed for eligibility according to the following inclusion criteria: (1) case-control design, (2) investigation of the association of IL-6 −174G/C polymorphism and susceptibility to IS, (3) providing sufficient data about the genotype frequencies of IL-6 genetic polymorphisms for calculating the value of odds ratio (OR) and 95% CI, and (4) full-text articles. Studies were excluded if one of the following criteria was fulfilled: (1) unrelated to IL-6 polymorphisms or IS (2) included reviews/comments/letters, (3) included case reports or case series, and (4) lack reusable data. If duplicate reports by the same authors or the same group of patients were found, the team included only the most complete study for pooled analyses.

### Data Extraction

Following PRISMA guidance (14), the following data were extracted from each eligible study independently by two reviewers; (Jie Chai and Xian-Ling Cao): first author name, publication year, study design, number of cases and controls, number of males and females, sample ethnicity, mean age of each group, allele frequencies, and genotype of IL-6 174G/C SNP gene polymorphisms in cases and controls. Discrepancies were resolved by discussion to reach consensus and arbitrated by a third person (Feng Lu) when necessary.

### Quality Assessment

The quality of methods for included studies was independently assessed by two reviewers; (Jie Chai and Xian-Ling Cao) using *Newcastle-Ottawa Scale (NOS)*. NOS uses a star rating system to assess quality and studies scores ranging from 0 to 9 stars (15, 16). Twelve of thirteen included studies had NOS ≥ 7, demonstrating good methodologic quality and a low risk of bias. Discrepancies between the two reviewers were resolved by a consensus agreement or by consulting the senior author (Feng Lu).

### Statistical Analysis

The strength of the association between IL-6 −174G/C(rs1800795) gene polymorphisms and IS risk was measured by ORs with 95% CIs for allele comparison (G vs. C), homozygote comparison (GG vs. CC) and (GC vs. CC), dominant (GG vs. GC + CC), hyper dominant (GG + CC vs. GC), and recessive (GG + GC vs. CC) models. The deviation of HWE in the control group was tested by the goodness-of-fit chi-square test. A *P*-value < 0.05 was considered statistically significant. The meta-analysis was performed using Stata (version 16). Statistical heterogeneity among studies was estimated using the *Q*-test and I^2^ statistics. Heterogeneity was acceptable as long as I^2^ ≤ 50%. A random-effects model was used to estimate the pooled log ORs and 95% CIs as heterogeneity reached a *P* < 0.1 or I^2^ > 50%.

## Results

### Literature Search

**Figure 1:**
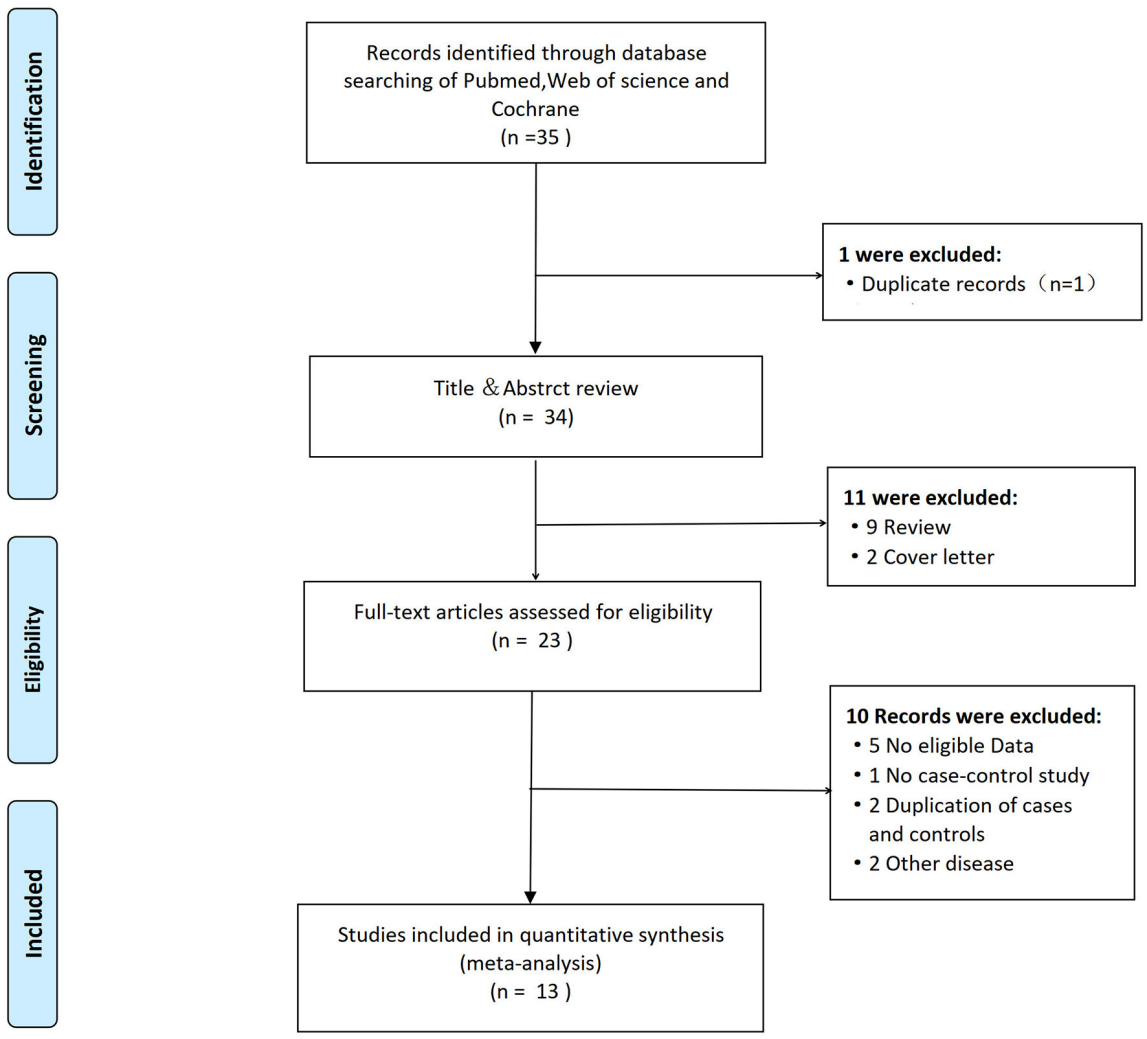
Flow diagram summarizing the search strategy for meta-analysis of 174G/C polymorphism of interleukin-6 (IL-6) and the risk of ischemic stroke.

Figure 1 displays the PRISMA flow diagram of the detailed literature screening process. A total of 35 articles, including one duplicate record, were found after a comprehensive literature search of PubMed, Web of Science, and the Cochrane central register of controlled trials. After carefully reviewing the abstracts of 34 non-duplicate studies, we excluded 11 studies as nine were review articles and two were cover letters. For the remaining 23 articles, five did not demonstrate eligible data, and one was excluded because it was not a case-control study (17). Two studies had overlapped data with duplication of cases and controls in Pola et al., Flex et al., Revilla et al., and Chamorro et al. (18–21). Two studies were about another disease. Finally, this meta-analysis included 13 eligible studies with an overall sample size ranging from 42 to 748 cases.

### Study Characteristics

Table 1 summarizes the essential characteristics of studies included in our meta-analysis, including the author, region, publication year, case/control, age, genotyping, HWE *P*-value, and quality score. These studies, which were enrolled from 2003 to 2016, encompassed 2,654 cases and 2,894 controls. Regarding patient's ethnicity, the 13 patients were from Italy (2), India (2), Australia (3), China (1), Ireland (1), Spain (1), Poland (1), Turkey (1), and Croatia (1). The study by Balcerzyk et al. (27) on IS in children revealed that the mean age of controls was <25 years, excluding studies without reference to an average age. While HWE *P*-value in the study by Tuttolomondo et al. was 0.001 (24).

**Table 1:** Main characteristics of studies included.

**References**	**Cases/controls**	**Region**	**Male:female ratio**	**Age (mean** **±** **S) [range]**		**Disease**	**Cases**			**Controls**			**HWE(*P* 值)**	**Quality score**
			**Cases Controls**	**Cases**	**Controls**		**GG**	**GC**	**CC**	**GG**	**GC**	**CC**		
Yan et al. (10)	65/47	Austral-ian	42:23/22:25	Median 78 [27–89]	Median 55 [21–95]	IS	14	26	25	14	23	9	0.94	8
Bazina et al. (22)	114/187	Croatian	79:35/70:117	Median 54	Median 55	IS	39	22	53	63	26	98	2.16	7
Ozkan et al. (7)	42/48	Turkey	21:21/17:31	63.57 ± 15.3	62.29 ± 12.6	IS	4	22	16	14	21	13	0.39	8
Chakraborty et al. (23)	100/120	Indian	69:31/83:37	54.0 ± 10.9 [20–82]	52.5 ± 9.8 [34-79]	IS	57	35	8	73	39	8	0.38	7
Tuttolomondo et al. (19)	96/48	Italian	45:51/16:32	71.9 ± 9.75	71.4 ± 7.45	IS	40	46	10	14	33	1	0.001	5
Balcerzyk et al. (18)	80/138	Polack	45:35/73:61	Mean 14.3 [2–25]	Mean 7.5 [0.25-18]	IS	21	43	16	40	76	22	0.16	7
Tong et al. (9)	748/748	Chinese	379:269/379:269	61.12 ± 9.98	60.21 ± 9.89	IS	747	1	0	743	5	0	0.93	7
Banerjee et al. (24)	176/212	Indian	113:63/143:69	58.6 ± 14.2 [16–95]	57.4 ± 8.8 [16–95]	IS/HS	123	53	0	156	52	4	0.89	8
Lalouschek et al. (25)	404/415	Austral-ian	257:147/253:162	NM [<60]	NM	IS/TIA	143	187	74	156	192	67	0.54	7
Chamorro et al. (17)	273/105	Spanish	191:82/62:43	67 ± 10	64 ± 10	IS	104	134	35	46	50	9	0.37	8
Flex et al. (15)	237/223	Italian	132:105/107:116	76.2 ± 9.4	76.1 ± 6.8	IS	100	115	22	56	99	68	0.10	8
Balding et al. (26)	105/389	Irish	63:42/226:163	Mean 69 [35-69]	Mean 37.1 [18–65]	IS	33	60	12	123	198	68	0.44	7
Greisenegger et al. (27)	214/214	Austral-ian	129:85/129:85	49.2 ± 8.7 [18–60]	NM [15–63]	IS/TIA	81	96	37	76	108	30	0.39	8

*IS, Ischemic Stroke; TIA, Transient Ischemic Attack; NM, not mentioned; LS, lacunar stroke; HS, Hemorrhagic Stroke; HWE, Hardy-Weinberg Equilibrium*.

### Results of Meta-Analysis

**Figure 2:**
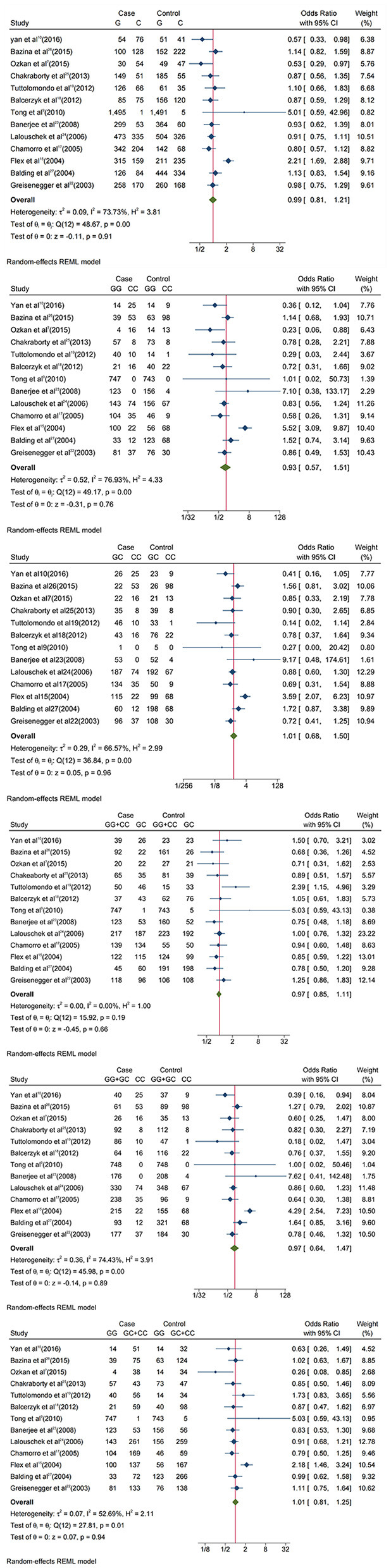
Forest plots of Interleukin-6 −174G/C(rs1800795) polymorphism and IS. The allelic comparison (G vs. C), homozygote comparison (GG vs. CC) and (GC vs. CC), hyperdominant (GG + CC vs. GC), recessive (GG + GC vs. CC,) and dominant (GG vs. GC + CC).

The results of meta-analysis are displayed in Figure 2; Table 2. IL-6 −174G/C(rs1800795) polymorphism has no correlation with IS susceptibility. OR and 95% CI for each model were as follows: G vs. C: 0.99 [0.81, 1.21]; GG + CC vs. GC: 0.97 [0.85, 1.11]; GG vs. GC + CC: 1.01 [0.81, 1.25]; GC vs. CC: 1.01 [0.68, 1.5]; GG vs. CC:0.93 [0.57, 1.51]; GG + GC vs. CC:0.97 [0.64, 1.47].

**Table 2:** Main results in the total and subgroup analysis.

**Study groups**	**G-allele vs. C-allele**		**GG vs. GC** **+** **CC**		**GG** **+** **GC vs. CC**		**GG vs. CC**		**GC vs. CC**		**GG** **+** **CC vs. GC**	
	OR(95% CI)	*P*	OR(95%CI)	*P*	OR (95% CI)	*P*	OR (95% CI)	*P*	OR (95% CI)	*P*	OR (95% CI)	*P*
Overall	0.99(0.81, 1.21)	0.91	1.01(0.81, 1.25)	0.94	0.97(0.64, 1.47)	0.89	0.93(0.57, 1.51)	0.76	1.01(0.68, 1.50)	0.96	0.97(0.85, 1.11)	0.66
Overall (Remove HWE *P* < 0.05)	0.98(0.79, 1.22)	0.86	0.98(0.78, 1.22)	0.83	1.02(0.68, 1.55)	0.91	0.97(0.59, 1,60)	0.90	1.07(0.73, 1.58)	0.73	0.94(0.82, 1.08)	0.38
**Subgroup analysis**												
By ethnicity	LogOR(95% CI)	*P*	LogOR(95%CI)	*P*	LogOR (95% CI)	*P*	LogOR (95% CI)	*P*	LogOR (95% CI)	*P*	LogOR (95% CI)	*P*
Asian	0.14(−0.44, 0.08)	0.16	−0.13(−0.51,0.24)	0.00	0.24(−0.40, 0.89)	0.45	−0.36(−1.57, 0.84)	0.17	−0.04(−0.72, 0.64)	0.45	−0.19(−0.51, 0.12)	0.38
European	0.14(−0.16, 0.45)	0.00	0.02(−0.72,0.77)	0.00	−0.17(−0.86, 0.52)	0.00	0.18(−0.58, 0.95)	0.00	0.20(−0.46, 0.85)	0.00	−0.08(−0.28, 0.12)	0.13
Oceanian	−0.11(−0.21, 0.19)	0.21	0.02(−0.16,0.21)	0.85	0.26(−0.02, 0.54)	0.27	−0.25(−0.56,0.07)	0.32	−0.26(−0.56, 0.04)	0.32	0.10(−0.11, 0.32)	0.46
By HWE *p* 值	LogOR(95% CI)	*P*	LogOR(95%CI)	*P*	LogOR (95% CI)	*P*	LogOR (95% CI)	*P*	LogOR (95% CI)	*P*	LogOR (95% CI)	*P*
*P* < 0.2	0.48(−0.21, 1.16)	0.02	0.73(0.38,1.08)	0.60	0.05(−3.03, 3.12)	0.00	0.41(−2.46, 3.29)	0.01	−0.18(−3.35, 2.99)	0.00	−0.04(−0.19, 0.10)	0.49
*P* ≥ 0.2	−0.08(−0.19, 0.02)	0.23	−0.10(−0.25,0.05)	0.50	−0.13(−0.36, 0.11)	0.23	−0.16(−0.39, 0.06)	0.23	−0.09(−0.34, 0.17)	0.24	0.30(−0.71, 1.32)	0.01

In subgroup analyses by ethnicity or HWE *P-*value, there was a statistically significant association between IL-6 −174G/C(rs1800795) polymorphisms and IS in the allele model (G vs. C: 1.15 [0.85, 1.57]), homozygote model (GG vs. CC: 1.2 [0.56, 2.58]) and (GC vs. CC: 1.22 [0.63, 2.35]), dominant model (GG vs. GC + CC:1.17 [0.83, 1.66]), and recessive model (GG + GC vs. CC: 1.19 [0.59, 2.37]) of European and in the allele model (G vs. C: 1.08 [0.61, 1.9]), homozygote model (GG vs. CC: 0.92 [0.23, 3.71]) and (GC vs. CC: 0.77[0.22, 2.74]), dominant model (GG vs. GC + CC: 1.27 [0.72, 2.25]), and recessive model (GG + GC vs. CC: 0.82 [(0.22, 3.04]) of HWE *P-*value < 0.2.

**Figure 3:**
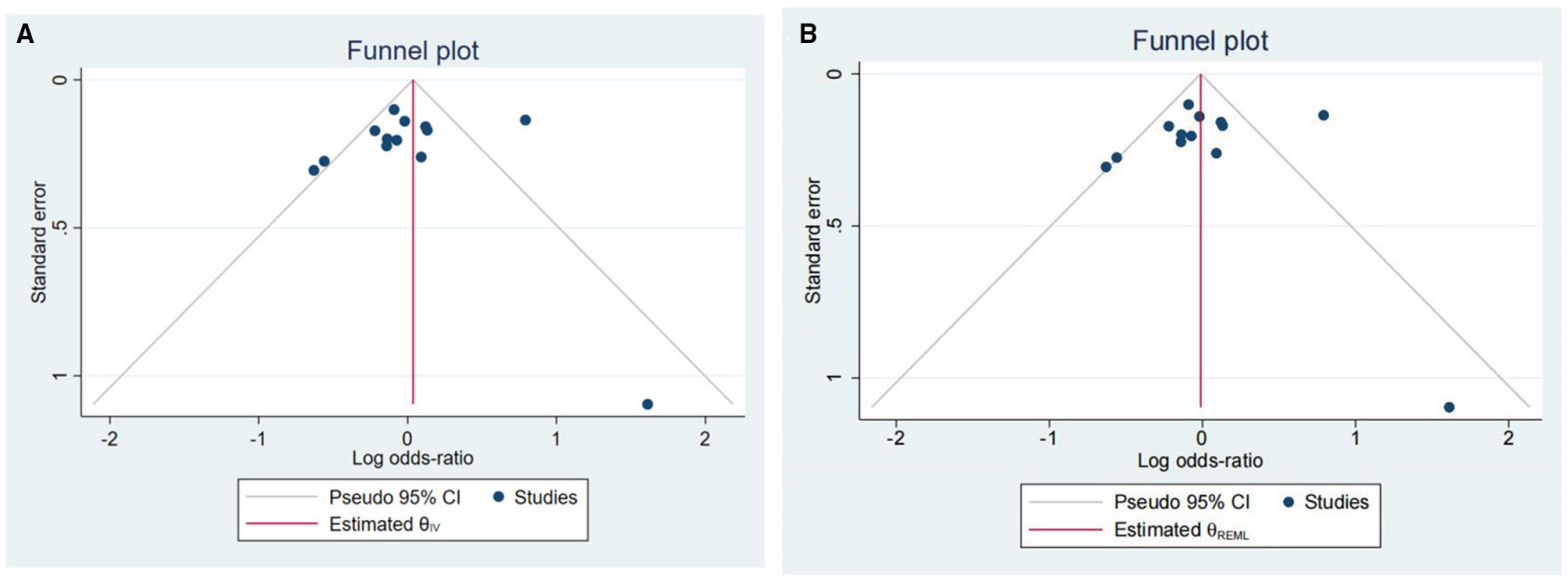
Funnel plots of publication bias. **(A)** nonparametric trim and **(B)** fill method corrected.

Figure 3 illustrates Begg's funnel plots estimating publication bias. The shape of funnel plots indicates significant relevant publication bias (Figure 3A). Therefore, we use the non-parametric trim and fill method to identify and correct the funnel asymmetry caused by publication bias (Figure 3B). The results show that OR value and 95% CI of the combined effect calculated by the random effect model is 0.99 [0.81, 1.21] after pruning, indicating that publication bias has an insignificant impact on our conclusion and that our conclusion is relatively reliable.

## Discussion

In this study, 13 independent studies were evaluated with 5,548 individuals, including 2,654 cases and 2,894 controls. Overall, this meta-analysis showed that in a large population, no association exists between −174G/C(rs1800795) and IS. Our results are consistent with Jin et al., but our number of studies is about twice that included in their survey after removing duplicate data. Moreover, we conducted a subgroup analysis on ethnicity and HWE values (25), demonstrating a significant difference among different populations, especially in the European population sample. Besides, the sub-group analysis of HWE indicated the same results. The risk of IS varies significantly by race and geography. IS is a major complication of atherosclerotic cardiovascular disease and involves complex biological processes and interaction pathways of non-genetic and genetic factors, causing a fairly high mortality (22, 23).

Given that animal and clinical trials demonstrated IS patients have elevated levels of pro-inflammatory cytokine IL-6, numerous studies were conducted to demonstrate the relationship between SNP −174G/C(rs1800795) in the promoter region of the IL-6 gene and IS but with different conclusions (21, 26, 28, 29). Although SNP −174G/C(rs1800795) is evidenced to be involved in IS pathophysiology, there is little evidence to date that SNP −174G/C(rs1800795) in the promoter region of the IL-6 gene is a risk factor. Banerjee et al. stated no significant differences in IL-6 −174G/C frequency between crude IS cases and controls in the Indian population sample (28). Meanwhile, Chakraborty et al. found that GC genotype is linked to increased mortality and a poorer outcome of the Indian population (30).

Moreover, Tong et al. revealed that IL-6 polymorphism at −174 is unlikely to significantly contribute to susceptibility or affect IS progression in either Han or Uyghur populations (12). On the contrary, in a study of 114 patients with IS and 187 healthy controls, Bazina et al. found that IL-6 −174G/C polymorphism can be employed as a candidate gene marker and risk factor for predicting the early onset of IS in the Croatian population sample (31). In contrast, Yan et al. revealed that the G allele of the IL-6 promoter 174 G/C polymorphisms protects against stroke (13). Furthermore, Ozkan et al. demonstrated that IL-6 gene polymorphism is associated with IS, both homozygous and heterozygous conditions, in patients of the south Marmara region of Turkey (10). However, Lalouschek et al. and Balding et al. both found that IS incidence was not significantly correlated with IL-6 polymorphism (29, 32).

This current study has a few limitations. First, we have included a study of IS in children. However, after excluding this study, the meta-analysis also showed no statistically significant difference. Second, we included a study in which HWE *P* < 0.05, but no significant difference was found after removing this study, and we conducted a subgroup meta-analysis. It should be noted that the small sample size of included studies may limit the generalizability of our findings. Finally, another potential limitation is that our meta-analysis was performed at a study level, implying the reuse of data provided in papers. As a result, it is evident that there is a publication bias between the studies included. Although we used additional methods to prove that this impact is negligible, we cannot completely ignore the potential impact of publication bias on our research results.

There is a high degree of heterogeneity in our meta-analysis. Using different methods to assess the potential sources of heterogeneity indicates that heterogeneity can almost be explained by national and ethnic differences. After careful reading and summarizing the articles, we found that the possibility of clinical heterogeneity is small. Due to slight differences in gene detection technology, there may be methodological heterogeneity. These results may indicate that our heterogeneity is mainly attributed to statistical and methodological heterogeneity. Our research findings require careful consideration, and larger-scale experiments are required to verify these findings.

In conclusion, although our study indicates that IL-6 −174G/C(rs1800795) polymorphism does not correlate with IS susceptibility, the subgroup analysis revealed that IL-6 −174G/C(rs1800795) polymorphism is linked to IS susceptibility in the European population sample. It also indirectly confirms that there are racial differences in IS susceptibility. However, further studies using a larger sample from multiple centers may enhance our study results.

## Data Availability Statement

The original contributions presented in the study are included in the article/supplementary material, further inquiries can be directed to the corresponding author/s.

## Author Contributions

JC and X-LC wrote this paper. FL revised this paper. All authors contributed to the article and approved the submitted version.

## Funding

This work was funded by Key Project of National Key R&D Program for Modernization of Traditional Chinese Medicine 2018 (No. 2018YFC1707402) and Business Construction of National Clinical Research Base of Traditional Chinese Medicine in 2016 (No. JDZX2015141).

## Conflict of Interest

The authors declare that the research was conducted in the absence of any commercial or financial relationships that could be construed as a potential conflict of interest.

## Publisher's Note

All claims expressed in this article are solely those of the authors and do not necessarily represent those of their affiliated organizations, or those of the publisher, the editors and the reviewers. Any product that may be evaluated in this article, or claim that may be made by its manufacturer, is not guaranteed or endorsed by the publisher.
